# Evaluation of primary wound healing and potential complications after perioperative infiltration with lidocaine without adrenaline in surgical incisions in dogs and cats

**DOI:** 10.1186/s13028-023-00686-x

**Published:** 2023-06-13

**Authors:** Erica Anna Gumpert Herlofson, Francesca Tavola, Karolina Siri Engdahl, Annika Filippa Bergström

**Affiliations:** 1 Department of Clinical Sciences, Faculty of Veterinary Medicine and Animal Science, Swedish University of Agricultural Sciences, Uppsala, Sweden; 2 Awake Djursjukhus, Hornsgatan 150A, Stockholm, 117 28 Sweden

**Keywords:** Lidocaine, Local anaesthesia, Multimodal analgesia, Thermography, Wound healing

## Abstract

**Background:**

Pre-emptive local analgesia with the use of lidocaine is practised increasingly in veterinary medicine as part of applied multimodal analgesia, despite its controversial impact on wound healing. The purpose of this prospective, randomised, double-blinded, placebo-controlled clinical study was to evaluate if preoperative subcutaneous infiltration of lidocaine has a negative impact on primary wound healing of surgical incisions. Fifty-two companion animals (3 cats and 49 dogs) were enrolled in the study. The inclusion criteria were as follows: American Society of Anaesthesiologists (ASA) score I or II, a minimum body weight of 5 kg, and a planned incisional length of at least 4 cm. Surgical incisions were infiltrated subcutaneously with lidocaine without adrenaline or NaCl (placebo). Follow-up questionnaires for owners and veterinarians and thermography of the surgical wound were used to assess wound healing. Antimicrobial use was documented.

**Results:**

There was no significant difference in either the total score or the individual assessment points between the treatment and the placebo group on the owner or the veterinary questionnaires in regard to primary wound healing (P > 0.05 for all comparisons). No significant difference was found between the thermography results of the treatment and placebo group (P = 0.78), and there was no significant correlation between the total score from the veterinary protocol and thermography results (Spearman’s correlation coefficient − 0.10, P = 0.51). Surgical site infections developed in 5/53 (9.4%) surgeries and its occurrence varied significantly between the treatment and the placebo group as all cases of infection were in the placebo group (P = 0.05).

**Conclusion:**

The results of this study indicate that lidocaine used as a local anaesthetic did not affect wound healing in patients with ASA scores I-II. The results suggest that lidocaine infiltration in surgical incisions can be safely used to reduce pain.

**Supplementary Information:**

The online version contains supplementary material available at 10.1186/s13028-023-00686-x.

## Background

Wound healing is an important physiological process in both human and animal patients to maintain skin integrity after injury, either by accident or intent procedure [1]. Surgery represents a traumatic insult to the body and is accompanied by a verifiable inflammatory response, dependent on the magnitude of the insult [2]. Local anaesthetic infiltration along the surgical incision is used to provide neuronal transmission blockade [3]. Infiltrating the surgical wound with local anaesthetic is increasingly used in the perioperative period; when infiltration analgesia is installed before surgical incision, it pre-emptively increases analgesic efficacy during and after surgery [4, 5]. The use of local anaesthetic agents as a part of multimodal anaesthesia prevents arise of nociceptive stimulus locally, enabling a reduction in the requirement of general anaesthetic agents resulting in better cardiovascular function [6].

Lidocaine remains the most versatile and widely used local anaesthetic in veterinary medicine because of its fast onset, moderate duration of effect, and moderate toxicity compared to other local anaesthetics. The reported onset and duration of plain lidocaine are approximately < 2 min and 1 h, respectively [6]. Lidocaine and other local anaesthetics have been investigated for their effect on wound healing in both in vitro and in vivo studies, but the results are equivocal [7]. Some studies performed using laboratory animals and testing tissue samples and tissue cultures reported that lidocaine delays wound healing by decreasing wound tension strength [8, 9], impairing lymphatic function [10], inhibiting the synthesis of collagen and glycosaminoglycan [11, 12], increasing cytotoxicity in fibroblast [13], and inhibiting lysophosphatidate signalling [14]. Other studies suggest that lidocaine does not affect wound healing [15, 16].

Although there is no validated protocol for owners evaluating surgical wounds, scoring of swelling, redness, and discharge has been reported successfully for wound healing evaluation by owners [17, 18].

Studies have been performed in both human [19, 20] and veterinary [21, 22] medicine using thermal imaging to successfully evaluate postoperative inflammation in wounds and to identify deviations from normal wound healing progression through healing phases [23–25]. Thermography uses emitted heat from a given source to make a visual image of the temperature depicted in colours visible to the human eye. The visual heat pattern, with lower temperatures depicted as blue-green and higher temperatures showing as orange-red, can then help the practitioner to identify asymmetries in heat emission that can serve as an indication of an ongoing and potentially pathological process in the area [25]. The method has been used in small animal studies with the intention to identify thoracolumbar disc hernia [26], muscle injury [27], and painful conditions in cats [28]. Thermal imaging is a very sensitive, no-contact examination technique and the imaging process presents no risk to the patient or the examiner through radiation [29]. Furthermore, there is no need to sedate the animal, which may be required for other diagnostic imaging techniques [26]. The greatest limitation is that although it is very sensitive, thermography is not very specific and therefore is best used together with other diagnostic tools [26].

In spite of the extensive use of perioperative subcutaneous infiltration of lidocaine, its impact on wound healing is still debated. Thus, the aim of this study was to evaluate the effect on primary wound healing of preoperative infiltration of local anaesthesia with plain lidocaine along the surgical incision, by assessing surgical wounds clinically and with thermography. The hypothesis was that preoperative lidocaine would not have a negative impact on wound healing at the time of clinical evaluation for suture removal (12–16 days after surgery) when full wound healing is anticipated. To the authors’ best knowledge, this is the first study describing the effect of preoperative infiltration of lidocaine on wound healing in a clinical veterinary setting.

## Methods

### Patient selection

The selected study population consisted of client-owned dogs and cats booked for surgery at the University Animal Hospital, Swedish University of Agricultural Sciences, Uppsala, Sweden between April 2016 to October 2016. Clients were asked to sign a consent form for participation in the study after receiving written and oral information. The study was approved by the Uppsala Animal Ethics Committee (C73/15).

Species, breed, sex, body weight, and age were registered. The surgeries were categorised into four groups: abdominal surgery with ventral midline incision, mastectomy, orthopaedic surgery, and other surgeries. Antibiotic treatment and surgical site infections (SSIs) were registered.

### Inclusion and exclusion criteria

Inclusion criteria were met for patients scheduled for clean or clean-contaminated surgery with an American Society of Anaesthesiologists (ASA) score of I or II, a body weight of at least 5 kg, and a planned incision estimated to exceed a length of 4 cm. Exclusion criteria were as follows: ASA score above II, systemic illness, immunosuppressive treatment, concurrent skin disease (e.g. atopy), and a history of prolonged wound healing. Patients were also excluded if they underwent major tumour resections, advanced reconstructive surgery, or had wounds in need of drainage given the increased risk for complications after these procedures [30, 31]. Carpal and tarsal arthrodesis were excluded, since postoperative cast or bandage and tension relieving techniques commonly used affect the possibility to evaluate the wound subjectively and with thermography.

At the time of discharge, all owners received standardised instructions regarding wound care and the use of an E-collar.

### Randomisation of treatment

A randomly selected envelope allocated patients to either the group administered lidocaine or the placebo group. The envelopes were drawn at admission and opened in the preoperative preparation room. Nurses not committed to the study performed both the selection and the opening of envelopes. The selected substance was administered by the nurse assigned to assist during surgery and was given in a standardised (sliding needle technique) and sterile manner before moving the patient to the operating table. Both the study manager and surgeon were blinded to the content of the envelope. Patients assigned to the treatment group received 0.25 mL Xylocaine (lidocaine without adrenaline 10 mg/mL) per incision cm whereas patients assigned to the placebo group were given corresponding volumes of NaCl. Injections were administered subcutaneously with a maximum dose of 5 mg lidocaine/kg.

### Follow-up assessment

The clients were asked to fill in a questionnaire regarding the wound five to seven days after surgery. The questionnaire included individual assessment points that were scored 0 = absent and 1 = present for the following parameters: pain, wound dehiscence, redness, swelling, and suppuration. A total score was calculated by summarising the individual scores, the maximum score of owner evaluation was 5.

For wound assessment, photographic documentation of the incision site and thermal imaging were performed by a veterinarian 12 to 16 days after surgery. Wounds were scored for SSI and pain (0 = absent, 2 present). The presence of seroma, suppuration, redness, swelling and wound dehiscence were scored as 0 = absent, 1 = minor, 2 = major. In addition, redness, swelling and wound dehiscence were given extra points, depending on the proportion of the wound that was affected, as 0 = absent (0–33% affected), 1 = minor (34–66% affected), 2 = major (67–100% affected). The maximum score of veterinary evaluation was 20 by summarising the individual scores.

Surgical site infection was diagnosed based on criteria from Centers for Disease Control and Prevention (CDC) guidelines for superficial incisional SSI [32]. In any case of wound healing complications, the owner was asked to contact the hospital, and the diagnosis of SSI as well as the decision to initiate antimicrobial therapy was made by a veterinarian at the hospital.

A Meditherm camera, IRIS 2000 was used for thermal imaging with a 25 deg FOV lens with a focus range from 5 cm to infinity. The patient was left in a draught-free and ambient examination room for 10 min to ensure acclimatisation before thermal imaging was performed. The positioning of the patients was adapted depending on the anatomical site of the wound and patient comfort when imaging was performed. Thermal imaging was performed with a distance of 30–45 cm between the patient and the camera, while the patient was standing or lying down.

The beginning and the end of the wound were marked and additional markers were strategically placed in non-linear wounds with non-transparent adhesive tape preventing heat emission from these areas to create an intentional artefact in the thermal image. Thermal images were processed using customised software developed by Meditherm Inc. from the original version of the WinTes3 program (Uppsala, Sweden, 2016).

A Casio Exilim HS, EX-ZR 1000 was used to take images of the surgical wounds in order to interpret the wounds in a standardised way. To standardise optical effects two distances, 10 and 15 cm, were attached to the camera when photographs were taken. Printed adhesive etiquettes for journal reference were attached to the distances and visible in photographs. A generalised photo of the entire area was also taken at a convenient distance in animals with long surgical incisions. In case of abnormal clinical scoring, close-ups of the areas of interest were taken as a point of reference to enable comparison between patients and wound assessments and assure uniform wound interpretation.

### Image processing

**Fig. 1:**
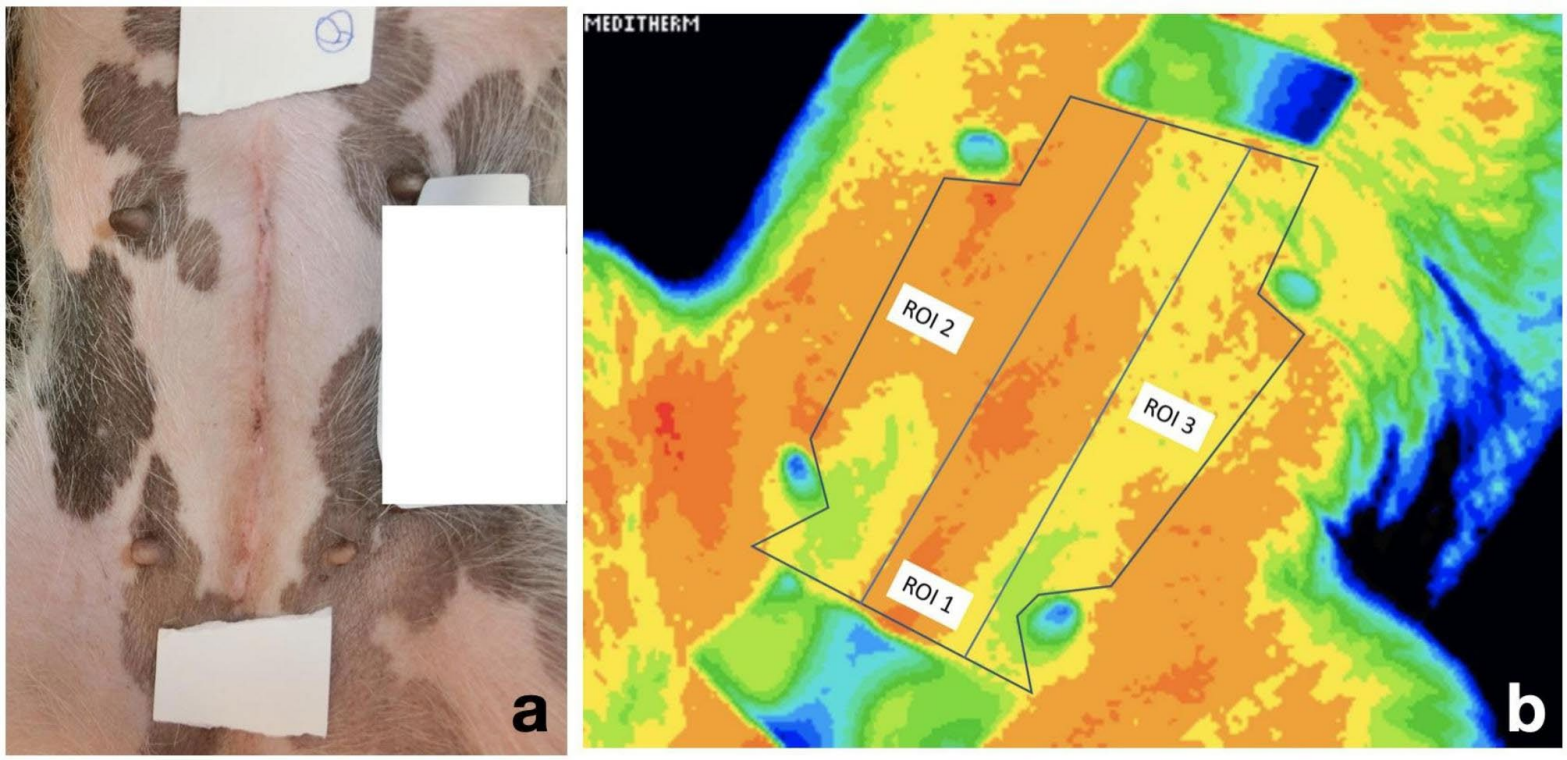
**a and b.** Photographic- and thermographic images of a ventral midline incision. **a**) Image of a ventral midline incision after ovariohysterectomy; the white tape marks the start and end of the surgical incision. **b**) Thermal image of the same patient. The white tape and teats are depicted in green-blue colours. Three regions of interest (ROI) are outlined, ROI 1 (wound area) in the middle, and ROI2 and ROI3 on each side of RO1. Note that teats are avoided in ROI2 and 3

Two-three regions of interest (ROI) were created depending on the anatomical site: ROI 1 included the surgical incision, and ROI 2 and ROI 3 (when possible to create) control areas. When the thermal images were processed, each image contributed the lowest, highest and mean temperature of that area (Fig. 1). To calculate the temperature difference between the wound and the control areas, the mean temperature of the control area/-s (ROI 2 and 3) was subtracted from the mean temperature of ROI 1 (surgical area).

### Statistical analysis

All statistical analyses were performed in R version 1.3.959. Categorical variables are described as numbers and percentages per category and continuous variables as mean (min–max, for normally distributed variables), or median (min–max, for non-normally distributed variables). P-values below 0.05 were considered to indicate statistical significance. The Shapiro-Wilk test was used to test the normality of continuous variables. Differences in demographic data (sex, body weight, and age), type of surgery, room temperature at thermography assessment, peri-operative antibiotic treatment, and occurrence of infection in the treatment and the placebo groups were tested with two-sided chi-squared test or Fisher’s exact test for categorical variables, and with Wilcoxon rank sum test for continuous variables.

Differences between the treatment and the placebo group in individual assessment points and total scores on the owner questionnaire and the clinical wound healing protocol were tested with Fisher’s exact test and Wilcoxon rank sum test. In addition, the results from the owner questionnaire were tested against infection with Fisher’s exact test. Differences in thermography scores between animals with and without nylon sutures present at thermography examination and between the treatment and placebo groups were tested with Student’s t-test. The correlation between the thermography results and the total inflammation score from the clinical wound healing protocol was tested with Spearman’s rank correlation coefficient.

## Results

The study population included 3 cats and 49 dogs, of which one dog was included twice as it had two surgeries performed at different time points, resulting in 53 included surgeries. There were 31 females and 21 males, the median age was 7 (1–13) years and the median body weight was 16.9 (5.4–60.1) kg. Three cat breeds, 30 dog breeds, and 8 mixed-breed dogs were included; the most common dog breeds were golden retriever, Jack Russell terrier, Shetland sheepdog, and Dachshund (3 of each breed). Of the surgeries, 20 cases were abdominal surgeries with ventral midline incisions, 13 cases mastectomies, 12 cases orthopaedic surgeries (tibial plateau levelling osteotomy, lateral fabellar suture, shoulder arthroscopy because of osteochondrosis, medial shoulder instability stabilisation, femoral fracture repair and femoral head and neck excision), and 8 cases other types of procedures. The group with other surgeries included four prescrotal castrations, two cryptorchid castrations with an inguinal approach, one removal of a mastocytoma localised on the lateral aspect of the proximal part of the hind limb, and one cutaneous mastocytoma localised over the sacral area. The lidocaine treatment group included 25/53 surgeries and the placebo group 28/53 surgeries. There were no significant differences in age (P = 0.585), gender (P = 0.738), body weight (P = 0.575), or type of surgery (P = 0.232) between the treatment and the placebo groups.

Surgical site infection developed in 5 surgeries (9.4%). The occurrence of SSIs varied significantly between the treatment and the placebo group as all cases of infection were in the placebo group (P = 0.05). The mean number of days from surgery to infection was 7.4 (3–16), and all SSI were treated with antibiotics. Peri- or postoperative antibiotic treatment for reasons other than SSI was used in 18 surgeries of which 6 cases were treated with antibiotics for reasons unrelated to surgery (See Additional file 1 for details). The number of surgeries that received peri- or postoperative antibiotics did not vary significantly between the treatment and placebo groups (P = 0.16).

The owner questionnaire was completed after five to seven days in 79.2% of the surgeries. The median total score from the owner questionnaire was 0.5 (0–3) in the treatment group and 1 (0–4) in the placebo group, and there was no significant difference in the total score or the individual assessment points between the groups (P > 0.05 for all comparisons). There was a significant association between the total score and SSI: the median total score for animals with SSI was 3.0 compared to 0.5 for animals without infection (P = 0.019).

Follow-up veterinary examination 12–16 days after surgery was performed in 96.2% of the surgeries. The median total score from the clinical wound healing protocol in the treatment group was 3 (0–8) and in the placebo group 2.5 (0–13). There was no significant difference in the total score or in the individual assessment points between the treatment and the placebo group (P > 0.05 for all comparisons).

Of the 51 surgical wounds assessed in the veterinary follow-up examination, 98.0% were examined with thermography. The median room temperature at the thermography examination was 21.2 °C (19.5-29.0 °C) and did not vary between treatment groups (P = 0.98). Eighteen surgical wounds had nylon sutures present at the thermography examination, and there was no significant difference in thermography results for surgical wounds with and without nylon sutures (P = 0.554). No significant difference was found between the thermography results from the treatment and placebo group (P = 0.776), and there was no significant correlation between the total score from the veterinary clinical wound healing protocol and the results from the thermography (Spearman’s correlation coefficient − 0.10, P = 0.505).

## Discussion

This study evaluated the effect of preoperative infiltration of local anaesthesia with lidocaine along the surgical incision on primary wound healing in dogs and cats. No significant association between the lidocaine administration and delayed wound healing was found by the time of suture removal, based on thermography and subjective wound evaluation.

Although local anaesthetics have been studied both regarding efficacy in providing pre-emptive analgesia in perioperative wound management [5] and potential effects on wound healing, the latter has exclusively been studied in a laboratory setting [8, 15, 33, 34]. Therefore, this study focused on a clinical setting to apply the results directly to patient management. We found no evidence that the use of lidocaine for local infiltration should be withheld in ASA I-II patients in clean or clean-contaminated wounds.

Superficial surgical site infection was the only factor not evenly distributed between the treatment and the placebo groups. Since all SSI cases occurred in the placebo group, local anaesthesia with lidocaine presented no increased risk of SSI in the current study. These results could be due to the antimicrobial effect of lidocaine against *Staphylococcus aureus, Escherichia coli*, and *Pseudomonas aeruginosa* that has been demonstrated in vitro and in experimental studies [35, 36].

The wounds were assessed by the owners 5 to 7 days after surgery and by a veterinarian 14 to 16 days after surgery. Despite the time frame not being standardised for each patient, a significant difference was not expected as the assessments were performed after the inflammatory phase of wound healing only 24–48 h apart. Additional evaluations in the early phase of wound healing could have been beneficial in the early detection of impaired wound healing.

Thermal images did not show a higher temperature in wounds with an increased total inflammation score on the clinical wound healing protocol, which is contrary to what was initially expected. It can be debated that the degree of inflammation was not significant enough to generate a difference in skin temperature compared to surrounding tissue. The control areas (ROI 2 and ROI 3) were close to ROI 1 with a potential risk of elevated temperature in the control areas closer to the wound area, but no temperature increase in control areas close to the wound area was noted. Every region had a high, low, and mean temperature area, supporting this. By using control areas close to the wound, the skin was, in general, similar in thickness and type. Importantly, the wound and control areas were clipped at the same time (before surgery was performed) ensuring comparability since the presence of fur changes the degree of heat emission. Repeated thermal imaging at different stages including the inflammatory phase of wound healing might aid detection of a window of screening more appropriate to identify potential wound healing complications. Further, it should be noted that when thermography was performed, all patients with SSI, with the exception of one, had already received antibiotic therapy, influencing the validity of the data. Room temperature may affect wound temperature and efforts were made to ensure a consistent room temperature. This was not possible in one case. However, the dog was not excluded since the measurements were evaluated to be normal with no signs of increased inflammatory scores or temperature.

Early detection of SSIs is crucial for successful treatment. In this study, an increased inflammation score from the owner protocol was associated with the development of SSI. This finding correlates with an earlier report that suggests that active assessment of surgical sites by owners provides clinicians with crucial help for the early identification of SSI [18].

This study was limited to evaluating short-term wound healing in a relatively small heterogeneous population sample. Only patients with the expected ability to heal normally were included, thus ASA III-V patients and surgeries prone to various types of healing complications were excluded. From an anaesthetic point of view, ASA III-V patients would largely benefit from local anaesthesia. However, no conclusions can be drawn from this study regarding the potential negative effects of lidocaine on wound healing in these patients, given the risk of reduced local perfusion. Further studies within this area are motivated. Surgical, incisional wound evaluation lacks validated protocols for companion animals, and the evaluation is subjective thus increasing the risk for bias despite blinding. However, similar protocols have been reported earlier [17, 18].

The possibility of statistical analysis lacking power must be considered due to the relatively small study population, as well as the variety of surgical techniques, wherein, for instance, changes in the effect of lidocaine on wound healing related to the location of the surgical site can be disguised.

It was not possible to monitor patient care provided by owners after discharge. Written and oral instructions were given, but the compliance of the owners is likely to differ. However, the compliance did most likely not differ between the treatment and the placebo group, as the owners were blinded.

## Conclusion

The use of lidocaine to perform presurgical incisional infiltration in dogs and cats with ASA status of I and II undergoing a clean or clean-contaminated surgery did not affect primary wound healing compared to placebo.

## Electronic supplementary material

Below is the link to the electronic supplementary material.

**Additional file 1**: Treatment period, antibiotics, and indication for the treatment of the 23 patients that received antibiotics

## Data Availability

The datasets analysed during the current study are available from the corresponding author upon reasonable request.
