# Supplementary material for: Evaluation of primary wound healing and potential complications after perioperative infiltration with lidocaine without adrenaline in surgical incisions in dogs and cats
Source: Acta Vet Scand. 2023 Jun 13;65:21. doi: 10.1186/s13028-023-00686-x (PMC10265759; doi:10.1186/s13028-023-00686-x)
Supplement: Supplementary file 1 — Additional file 1: Treatment period, antibiotics, and indication for the treatment of the 23 patients that received antibiotics [file 13028_2023_686_MOESM1_ESM.docx]

Additional file 1. Treatment period, antibiotics, and indication for the treatment of the 23 patients that received antibiotics

| **ID** | **Treatment period** | **Treatment duration (days)** | **Antibiotics** | **Study group** | **Indication** |
| --- | --- | --- | --- | --- | --- |
| 1 | Peri-and Post-OP | 1/* | Amoxicillin | Lidocaine | Prophylaxis/SSI |
| 2 | Peri- and post-OP | 2 | Amoxicillin | Placebo | Prophylaxis |
| 3 | Peri-OP | 1 | Ampicillin | Placebo | Prophylaxis |
| 4 | Peri-OP | 1 | 1^st^ gen cephalosporin | Lidocaine | Prophylaxis |
| 5 | Peri-OP | 1 | Sulphonamide + trimethoprim | Placebo | Unrelated to surgery |
| 6 | Post-OP | 8 | Amoxicillin | Lidocaine | Unrelated to surgery |
| 7 | Peri- and post-OP | 12 | 1^st^ gen cephalosporin | Lidocaine | Prophylaxis** |
| 8 | Peri- and post-OP | 8 | Ampicillin + amoxicillin | Placebo | Unrelated to surgery |
| 9 | Peri-OP | 1 | 1^st^ gen cephalosporin | Placebo | Prophylaxis |
| 10 | Peri-OP | 1 | 1^st^ gen cephalosporin | Placebo | Prophylaxis |
| 11 | Post-OP | 14 | Amoxicillin + Clindamycin | Lidocaine | SSI |
| 12 | Peri- and post-OP | 2 | Amoxicillin | Placebo | Unrelated to surgery |
| 13 | Post-OP | 10 | Amoxicillin | Lidocaine | SSI |
| 14 | Post-OP | 5 | Amoxicillin | Lidocaine | SSI |
| 15 | Peri-OP | 1 | Ampicillin | Lidocaine | Prophylaxis |
| 16 | Post-OP | 6 | Amoxicillin | Lidocaine | SSI |
| 17 | Post-OP |  | Metronidazole | Placebo | Unrelated to surgery |
| 18 | Peri-OP | 1 | 1^st^ gen cephalosporin | Placebo | Prophylaxis |
| 19 | Peri-OP | 1 | 1^st^ gen cephalosporin | Lidocaine | Prophylaxis |
| 20 | Peri-OP | 1 | 1^st^ gen cephalosporin | Lidocaine | Prophylaxis |
| 21 | Peri-OP | 1 | 1^st^ gen cephalosporin | Lidocaine | Prophylaxis |
| 22 | Peri-OP | 1 | 1^st^ gen cephalosporin | Placebo | Prophylaxis |
| 23 | Post-OP | 7 | Ampicillin + Amoxicillin + Fluoroquinolone | Placebo | Unrelated to surgery |
| *Treatment for SSI started after the follow-up period  **The patient had severe swelling and pain the day after orthopedic surgery and received treatment for suspected deep surgical site infection  *Gen* generation, *OP* operative, *SSI* surgical site infection | | | | | |
